# Knowledge and attitudes of Irish GPs towards abortion following its legalisation: a cross-sectional study

**DOI:** 10.3399/bjgpopen19X101669

**Published:** 2019-12-11

**Authors:** Raymond O'Connor, Jane O'Doherty, Michael O'Mahony, Eimear Spain

**Affiliations:** 1 Senior Research Fellow, Graduate Entry Medical School, University of Limerick, Limerick, Republic of Ireland; 2 Research Assistant, Graduate Entry Medical School, University of Limerick, Limerick, Republic of Ireland; 3 Counsellor and Therapist, Student Services, University of Limerick, Limerick, Republic of Ireland; 4 Senior Lecturer, School of Law, University of Limerick, Limerick, Republic of Ireland; 5 Senior Lecturer, Graduate Entry Medical School, University of Limerick, Limerick, Republic of Ireland

**Keywords:** Sexual health, Postgraduate education, Ethics, Pregnancy, unwanted, Ireland, Abortion, induced

## Abstract

**Background:**

In May 2018, the Irish Constitution was changed following a referendum allowing termination of pregnancy by abortion. It is envisaged that the majority of terminations will be by medical abortion and will take place in general practice before 12 weeks gestation.

**Aim:**

To elicit attitudes and level of preparedness of Irish GPs to provide medical abortion services.

**Design & setting:**

Cross-sectional study of 222 GPs who were associated with the University of Limerick Graduate Entry Medical School (GEMS) and GP training programme.

**Method:**

An anonymous online questionnaire was distributed via email. Reminders were sent 2 and 4 weeks later.

**Results:**

The response rate was 57.2% (*n* = 127/222). Of the responders, 105 (82.7%) had no training in this area, with only 4 (3.1%) indicating that they had sufficient training. Nearly all responders (*n* = 119, 93.7%) were willing to share abortion information with patients. Just under half of responders (*n* = 61, 48.0%) would be willing to prescribe abortion pills, with 47 (37.0%) unwilling to do so. Only 53 (41.7%) responders believed that provision of abortion services should be part of general practice, with 52 (40.9%) saying that it should not. As to whether doctors should be entitled to a conscientious objection but should also be obliged to refer the patient, 92 (72.4%) responders agreed. Over two-thirds of responders (*n* = 89, 70.1%) felt that necessary patient support services are not currently available.

**Conclusion:**

There is a lack of training and a considerable level of unwillingness to participate in this process among Irish GPs. There is also a perceived lack of patient support services for women experiencing unwanted pregnancy. It is incumbent upon state and professional bodies to address these issues.

## How this fits in

Medical abortion administered by GPs was introduced in Ireland in January 2019. Currently, there is significant resistance to the provision of this service in primary care, although most GPs are willing to provide information on abortion to their patients. The vast majority of Irish GPs lack training in the provision of abortion services. Such deficiencies require careful consideration and monitoring of the service for any resultant adverse outcomes.

## Introduction

Few areas of mainstream medical practice are as controversial as abortion, particularly for those who believe that it involves the extinction of a human life. It may be carried out medically with the anti-progesterone drug mifepristone, followed by misoprostol, a prostaglandin analogue.^1^ Until recently, abortion was illegal in Ireland unless there was a threat to the life of the woman.^2,3^ Beyond this, medical practitioners were limited to providing information on abortion services lawfully available in other states.^4^ In a referendum held in May 2018, the Irish people voted to remove Article 40.3.3, which prohibited abortion in all but the most limited of circumstances,^5^ from the Irish Constitution.

This referendum was preceded by a number of judgments in international tribunals on this issue, including the 2016 case of Mellet versus Ireland.^6^ In this case, the United Nations Human Rights Committee found that Amanda Mellet had been subjected to cruel, inhuman, and degrading treatment, to discrimination, and to an arbitrary (and therefore unlawful) interference with her right to privacy when she was forced to choose between carrying her foetus to term, knowing that it would not survive following a diagnosis of trisomy 18, or seeking an abortion abroad. Following consideration by a representative citizens’ group (the Citizens’ Assembly)^7^ and a parliamentary committee (the Joint Committee on the Eighth Amendment),^8^ it was decided to put the issue to the people in a constitutional referendum. A general scheme of a bill to regulate the termination of pregnancy^9^ was published in advance, which outlined the proposed abortion regime and provides for terminations without cause up to 12 weeks (section 12). After this limit, terminations will be available if there is a risk to the life or of serious harm to the health of a woman, or if there is present a condition affecting the foetus that is likely to lead to the death of the foetus either before, or within 28 days of, birth (sections 9, 10, 11, and 12). The referendum was decisive, with 66.4% voting in favour of repeal of the eighth amendment. Following the referendum result, legislation to regulate abortion in line with the proposals published before the referendum was introduced and became effective in January 2019.^9^

It is expected that GPs will provide medical abortion services in the majority of cases and will be very involved in all cases up to 12 weeks gestation.^10^ This marks a huge change for GPs in Ireland, who have until now been guided by the Irish Medical Council ethical guidelines prohibiting abortion with rare exceptions.^11^ GPs may also be uncomfortable with abortions sought for largely socioeconomic reasons.^12,13^

The aim of this research was to seek GPs' views on the provision of medical abortions in Ireland following repeal of Article 40.3.3 of the Constitution. The authors asked participants: (1) whether GPs would be prepared to share information on abortion with their patients; (2) whether they feel that necessary services, such as counselling, are available to women with unwanted pregnancies who might attend their practices; (3) whether they would be prepared to prescribe appropriate medication to bring about medically induced abortion; (4) what their views are regarding those who have a CO to issuing such prescriptions; and, (5) what level of training and experience they have in this area of practice.

## Method

This was a cross-sectional, questionnaire-based study of Irish GPs. Questions were developed based on the stated aims of the study. Closed questions were used for the most part, with options for free text responses allowing GPs to provide qualitative information in response to two questions. Data were collected pertaining to demographic details; willingness to share information about abortion; experience of sharing of such information; opinions on availability of services for women seeking advice on or having had an abortion; willingness to prescribe abortion pills; level of training; opinion on whether provision of abortion should be part of general practice; and opinion on the rights of doctors who have a CO. Two open-ended questions on the type of experience responders had in this area and what further training GPs require were also included. The survey was piloted among 9 GPs who were affiliated with the medical school. Following this, minor changes to the wording of the survey were made to aid clarity. The full questionnaire is available as supplementary Box S1.

An email inviting GPs to take part in the survey was circulated via an independent gatekeeper (GEMS research administrator) to 222 GPs. The email contained an information sheet, a link to the online survey, and a consent form. Any identifying information about participants in the survey was removed before analysis. The software used for the survey was surveymonkey. This platform permitted only one survey to be returned per participant. The survey was circulated on 2 July 2018, with two reminders sent on 17 July and 30 July 2018.

### Selection of study participants

GPs who were affiliated with GEMS and recent graduates of the University of Limerick Specialist Training Programme in General Practice were sent an invitiation to participate in the study. These GPs were working in three out of Ireland’s four health regions (Dublin Mid Leinster, South, and West) and were broadly representative of the national profile (Supplementary Box 1).^14^

### Data analysis

The data was analysed using numeric descriptive statistics. Answers to the two open-ended questions were uploaded to NVivo (version 11) and two authors used thematic analysis, as described by Braun and Clarke, to analyse the data gathered.^15^

## Results

The overall response rate to the survey was 57.2% (*n* = 127/222). All questionnaires were completed fully, with the exception of open question 11 (*’* *What further training* *,* *if any* *,* *do GPs require? Please explain* *‘*), which was answered by 120 (94.5%) responders.

Of the total responders, 48.8% (*n* = 62) were male and 51.2% (*n* = 65) female. While the location of responders closely matched those surveyed and that of GPs currently working in Ireland,^16^ age and sex analysis showed that responders were more likely to be female and younger. This is shown in supplementary Tables S1, S2, and S3.

The answers to the first four questions are summarised in Table 1. The vast majority of responders were willing to share information about abortion in other jurisdictions with their patients; 60.6% (*n* = 77) had actually done so in the previous 12 months. Over 70% (*n* = 89) of responders felt that necessary support services, such as counselling, were not currently available to women considering, or who have already had, an abortion.

**Table 1.** Answers to questions 4–7 (relating to information and services) (*n* = 127)

Question	**Yes, *n* (%)**	**No, *n* (%)**
Would you be willing to share information about abortion to your patients?	119 (93.7)	8 (6.3)
Have you shared information about abortion with one of your patients in the last 12 months?	77 (60.6)	50 (39.4)
Do you feel that there are necessary services, such as counselling, available to women who might attend your practice looking for information or advice on having an abortion?	38 (29.9)	89 (70.1)
Do you feel that there are necessary services, such as counselling, available to women who might attend your practice having had an abortion?	31 (24.4)	96 (75.6)

In this area of practice, 105 (82.7%) responders had no training, with only 4 (3.1%) answering that they had sufficient formal training. The responses to this question are summarised in Table 2.

**Table 2.** Answers to question 9: ‘What type of training do you have in this area of practice?’ (*n* = 127)

**None** **,** ***n*** **(%)**	**Some formal training** **,** ***n*** **(%)**	**Sufficient formal training undertaken to give information to patients and prescribe abortion pills** **, *n* (%)** ****
105 (82.7)	18 (14.2)	4 (3.1)

In answer to the question *‘* *Would you be willing to prescribe* *“a* *bortion* *p* *ills* *“ to bring about medically induced abortion before* *12* *weeks* *gestation?* *‘*, 38 (29.9%) responded ‘Yes’, 47 (37.0%) responded ‘No’, 19 (15.0%) responded ‘Unsure’, and 23 (18.1%) responded that they would ‘In some circumstances’.

When asked whether they believed that the provision of abortion pills should be part of general practice, 53 (41.7%) responded ‘Yes’, 52 (40.9%) ‘No’, and the remaining 22 (17.3%) ‘Unsure’. The responses to the question *‘* *Do you think that doctors who do not wish to be part of this programme should be entitled to a CO but they are obliged to refer the woman to a participating doctor?* *‘*, showed 92 (72.4%) to be in favour, 20 (15.7%) against, and 15 (11.8%) unsure.

The question *’* *Do you think that doctors who do not wish to be part of this programme should be entitled to a CO whereby they have no* *obligation or* *role in arranging or participating in the process?* *‘* found that 66 (52.0%) responded ‘No’, 44 (34.6%) ‘Yes’, and 17 (13.4%) ‘Unsure’ (Table 3).

**Table 3.** Answers to questions 8, 12, 13, and 14 (relating to the provision of abortion services in general practice) (*n* = 127)

**Question and number**	**Yes, *n* (%)**	**No, *n* (%)**	**Unsure, *n* (%)**	**In some circumstances, *n* (%)**
8. Would you be willing to prescribe ‘abortion pills’ to bring about medically induced abortion before 12 weeks gestation	38 (29.9)	47 (37.0)	19 (15.0)	23 (18.1)
12. Do you think that the provision of abortion pills should be part of general practice?	53 (41.7)	52 (40.9)	22 (17.3)	N/A
13. Do you think that doctors who do not wish to be part of this programme should be entitled to a CO but they are obliged to refer the woman to a participating doctor?	92 (72.4)	20 (15.7)	15 (11.8)	N/A
14. Do you think that doctors who do not wish to be part of this programme should be entitled to a CO whereby they have no obligation or role in arranging or participating in the process?	44 (34.6)	66 (52.0)	17 (13.4)	N/A

N/A = not applicable. CO = conscientious objection.

The opinions of the 127 responders on their experience in this area of practice were divided into six groups (Table 4); 53 (41.8%) reported having experience; however, the second largest group of 46 (36.2%) reported having no experience or training in the area.

**Table 4.** Answers to question 10: ‘What type of experience do you have in this area of practice?’ (*n* = 127)

A: Experience in general practice dealing with abortion issues and crisis pregnancy, *n* (%)	B: Experience or training abroad (including in the UK), *n* (%)	C: Experience working in family planning services or Obs/Gyn, *n* (%)	D: Very little, *n* (%)	E: No experience, *n* (%)	F: Other, *n* (%)
53 (41.8)	12 (9.4)	7 (5.5)	6 (4.7)	46 (36.2)	3 (2.4)

Obs/Gyn = Obstetrics and Gynaecology.

When questioned on what further training, if any, GPs require, 120 (94.5%) participants responded. These results are summarised in Table 5, with the majority looking for practical training (66, 55.0%).

**Table 5.** Answers to question 11: ‘What further training, if any, do GPs require? Please explain.’ (*n* = 120)

A: Practical training, *n* (%)	B: Guidelines, *n* (%)	C: Educational courses, *n* (%)	D: Other, *n* (%)
66 (55.0)	11 (9.2)	27 (22.5)	16 (13.3)

The practical training suggested by responders within the free-text box included training in blood tests to establish pregnancy in patients, the use of ultrasound to date pregnancies, training on the pharmacological makeup of abortion pills, training in aftercare issues, and referral pathways for patients if required. Responders stated that they wanted best practice guidelines developed, informed by guidelines from countries where abortion is currently available legally (information is available from the authors on request). The most popular form of training suggested was a full day workshop (17, 14.5%), while 6 (5%) responders suggested an online educational module.

The remaining 16 (13.3%) responses were categorised as ’other’ due to the nature of their comments. These included the responder’s unwillingness to be involved in termination of pregnancies, their objection to the proposed GP led service for abortions in Ireland, and not believing that abortion is an appropriate service in general practice. Information is available from the authors on request.

## Discussion

### Summary

To the authors’ knowledge, this is the first such survey to have taken place in Ireland since the legalisation of abortion.^17^ The results indicated mixed views on the provision of abortion services by GPs in Ireland. Less than half of responders (*n* = 53, 41.7%) believed that abortion service should be part of general practice, with a further 17.3% (*n* = 22) indicating that they were unsure if the service should be provided in this context. While almost half of those surveyed indicated a willingness to prescribe abortion pills (*n* = 61, 48.0%), over a third would not do so (*n* = 47, 37.0%). The majority of responders were willing to share abortion information with their patients (*n* = 119, 93.7%) and many had already done so (*n* = 77, 60.6%); however, a majority also felt that necessary services, such as counselling, are not available for women with unwanted pregnancies (*n* = 89, 70.1%). Since the introduction of the abortion service in January 2019, the Irish Health Services Executive has set up counselling services for women with unwanted pregnancies both by telephone and online (but not face-to-face) via the My Options service.^18^

There is a considerable lack of experience and training among Irish GPs in this area. Over 40% (*n* = 52) of responders reported having very little or no experience in the area (Table 4), 82.7% (*n* = 105) reported that they had received no training, and only 3.1% (*n* = 4) reported that they had sufficient training to provide a service (Table 2). Between December 2018 and March 2019 the Irish College of General Practitioners (ICGP) has provided training to 208 GPs through four workshops, with a further two workshops planned in April and May 2019. These figures amount to less than 5% of the total GP population. At the time of writing, over 600 health professionals (including GPs, practice nurses, and midwives) have attended training with the Southern Taskgroup on Abortion and Reproductive Topics training meetings since 1 January 2019.^19^ Furthermore, the ICGP published *I* *nterim* *C* *linical* *S* *upport* *G* *uidelines* in January 2019.^20^

At the time of writing (18 July 2019), 335 GPs have signed a contract to provide abortion services (O'Brien, personal communication, 2019); however, the overall number of GPs receiving training is problematic given the low levels of experience and training, and the desire for further education indicated by participants in this study.

The issue of the availability of an exemption for those with a CO, and the extent of that exemption, has been controversial in Ireland.^21^ The majority of responders believed that those who do not wish to be part of the process should be entitled to a CO, but should also be obliged to refer a woman to a participating doctor (*n* = 92, 72.4%).

### Strengths and limitations

This study had many strengths. The fact that it was short and anonymous encouraged a fair response rate of 57%, which is high for a GP survey.^22^ All of the study responders were actively practising GPs. The location of the responders was nationally representative (supplementary Table S1) and responders came from three out of the four healthcare regions in Ireland.

The age and sex of responders were not nationally representative (supplementary tables 2 and 3). Responders were more likely to be younger and female than the national profile; however, it is likely that young female patients with unwanted pregnancies will tend to seek out young female doctors, therefore the sample may actually be more representative for the GPs seen by these young women than a traditionally representative sample, and so this is a strength rather than a limitation.

One limitation of the study was that the authors only surveyed GPs who were affiliated with GEMS and recent graduates of the University of Limerick Specialist Training Programme in General Practice. It is possible that GPs without such an affiliation might have responded differently. It is possible, though unlikely, that the same GP could have answered multiple questionnaires from different internet protocol (IP) addresses. Furthermore, it is also possible that GPs who were less comfortable with electronic media were less likely to answer the questionnaire; however, as over 94% of Irish GPs are able to work with computerised records,^16^ this is very unlikely.

### Comparison with existing literature

Patient access to abortion information is considered essential, as all women need evidence-guided information in order to make a fully informed decision in the case of unwanted pregnancy.^23^ Of the total responders, 60.6% (*n* = 77) answered that they had shared information about abortion with one of their patients in the previous 12 months, compared to 45% in a 6-month period in an earlier Irish survey,^24^ indicating that women appear to be engaging with their GPs in recent years when considering accessing an abortion in another country.

Recently published research involving women who had either travelled abroad to access abortion in a clinic or had self-managed a medical abortion at home in Ireland using online telemedicine, suggested that irrespective of the pathway chosen, women experience a lack of pre- and post-abortion support in the Irish healthcare system.^25^ Waiting times for these services may extend to several weeks though this may be aided by the recently introduced My Options service.^18^ Guidelines in other jurisdictions state that women should have access to objective information and counselling about their pregnancy options in the case of unwanted pregnancy.^26^ While some studies show that women may experience mental health problems following abortion,^27–30^ others have challenged this.^31,32^ In any case, support techniques for a more informed choice are needed.^33^

This survey pointed to a significant minority of GPs who were not willing to prescribe abortion pills and/or did not feel that this service should be provided as part of general practice. An earlier Irish study showed that 10% of responders were of the view that abortion can never be allowed and 25% took the view that it can only be allowed in limited circumstances.^24^ The authors do not know if the reluctance on the part of responders to this study is ideological, due to a lack or training and/or expertise, or linked to the feeling that this service should not be provided in already overstretched GP practices, or for other reasons.

There are many barriers to the provision of first trimester abortion services, which include moral opposition, lack of training, busy physicians, staff harassment, and insufficient resources,^34^ all of which may contribute to a reluctance to provide first trimester abortion services. The World Health Organization supports the provision of abortion services at primary care level;^35^ however, evidence of support for a GP-led abortion service internationally is inconclusive. It has been argued that GPs are ideally placed to provide a community-led abortion service, particularly in rural and regional communities, a view supported by a qualitative study of 15 experts in abortion service provision conducted in Australia,^36^ though a descriptive-interpretive qualitative study of 32 Australian GPs found that some saw abortion as beyond the scope of their practice.^37^ Some GPs had religious or moral objections; others regarded abortion provision as complicated and difficult, and while some GPs expressed interest in abortion provision, they were concerned about stigma and the impact it may have on perceptions of their practice and the views of colleagues.^37^ A qualitative study of 30 obstetricians/gynaecologists found that the stigma and ideological contention surrounding abortion serve as barriers to the integration of abortion into medical practice.^38^ Alternatives to a GP-led service include primary care abortion clinics^39^ and abortion services supplied by non-doctor, ’mid-level’ healthcare providers.^40^

This research showed strong support for respecting CO in GP practices in Ireland. Chavkin *et al* argue that the ingredients that appear necessary for a functional health system that guarantees access to abortion while still permitting CO include clarity about who can object and ready access by mandating referral.^41^

### Implications for research and practice

The immediate implications for practice are the need for education courses for GPs on the provision of abortion services. The provision of integrated training in reproductive health^42^ and the provision of an elective advanced training and leadership programme for senior residents in family medicine^43^ have been shown to be effective.

Beyond the legal changes which have been implemented following the societal conversation on abortion in Ireland in recent times, there are many conversations on public policy on reproductive health which are currently being grappled with. The Citizens’ Assembly,^7^ the influential body comprising 99 citizens randomly selected to be broadly representative of the Irish electorate established to consider some of the most important issues facing Ireland’s future, including access to abortion, went beyond recommending legal changes to make some significant policy recommendations. Some of these recommendations have significant implications for general practice, including improved access to *’* *family planning services, contraception, perinatal hospice care* […] *to counselling and support facilities for pregnant women both during pregnancy and, if necessary, following a termination of pregnancy, throughout the country*’.^7^ The Joint Committee on the Eighth Amendment of the Constitution subsequently recommended that a scheme for the provision of free contraception be introduced, leading the Minister for Health to establish a Working Group on Access to Contraception in 2019 to consider the policy, regulatory, and legislative issues relating to better access to contraception.^8^ Should the government act to implement these policy recommendations, the landscape for GPs providing reproductive health care for women in Ireland will change dramatically.

The authors plan to repeat the study at the end of 12 months to reassess attitudes, knowledge, and (now) experience of GPs with providing the service. The authors also plan to assess centrally collected statistics and to study women’s experience of medical abortion in Ireland.
